# Conformational Changes Underlying Pore Dilation in the Cytoplasmic Domain of Mammalian Inward Rectifier K^+^ Channels

**DOI:** 10.1371/journal.pone.0079844

**Published:** 2013-11-11

**Authors:** Atsushi Inanobe, Atsushi Nakagawa, Yoshihisa Kurachi

**Affiliations:** 1 Department of Pharmacology, Graduate School of Medicine, Osaka University, Suita, Osaka, Japan; 2 Center for Advanced Medical Engineering and Informatics, Osaka University, Suita, Osaka, Japan; 3 Laboratory of Supramolecular Crystallography, Institute for Protein Research, Osaka University, Suita, Osaka, Japan; Sackler Medical School, Tel Aviv University, Israel

## Abstract

The cytoplasmic domain of inward rectifier K^+^ (Kir) channels associates with cytoplasmic ligands and undergoes conformational change to control the gate present in its transmembrane domain. Ligand-operated activation appears to cause dilation of the pore at the cytoplasmic domain. However, it is still unclear how the cytoplasmic domain supports pore dilation and how alterations to this domain affect channel activity. In the present study, we focused on 2 spatially adjacent residues, i.e., Glu236 and Met313, of the G protein-gated Kir channel subunit Kir3.2. In the closed state, these pore-facing residues are present on adjacent βD and βH strands, respectively. We mutated both residues, expressed them with the m_2_-muscarinic receptor in *Xenopus* oocytes, and measured the acetylcholine-dependent K^+^ currents. The dose-response curves of the Glu236 mutants tended to be shifted to the right. In comparison, the slopes of the concentration-dependent curves were reduced and the single-channel properties were altered in the Met313 mutants. The introduction of arginine at position 236 conferred constitutive activity and caused a leftward shift in the conductance-voltage relationship. The crystal structure of the cytoplasmic domain of the mutant showed that the arginine contacts the main chains of the βH and βI strands of the adjacent subunit. Because the βH strand forms a β sheet with the βI and βD strands, the immobilization of the pore-forming β sheet appears to confer unique properties to the mutant. These results suggest that the G protein association triggers pore dilation at the cytoplasmic domain in functional channels, and the pore-constituting structural elements contribute differently to these conformational changes.

## Introduction

**Figure 1:**
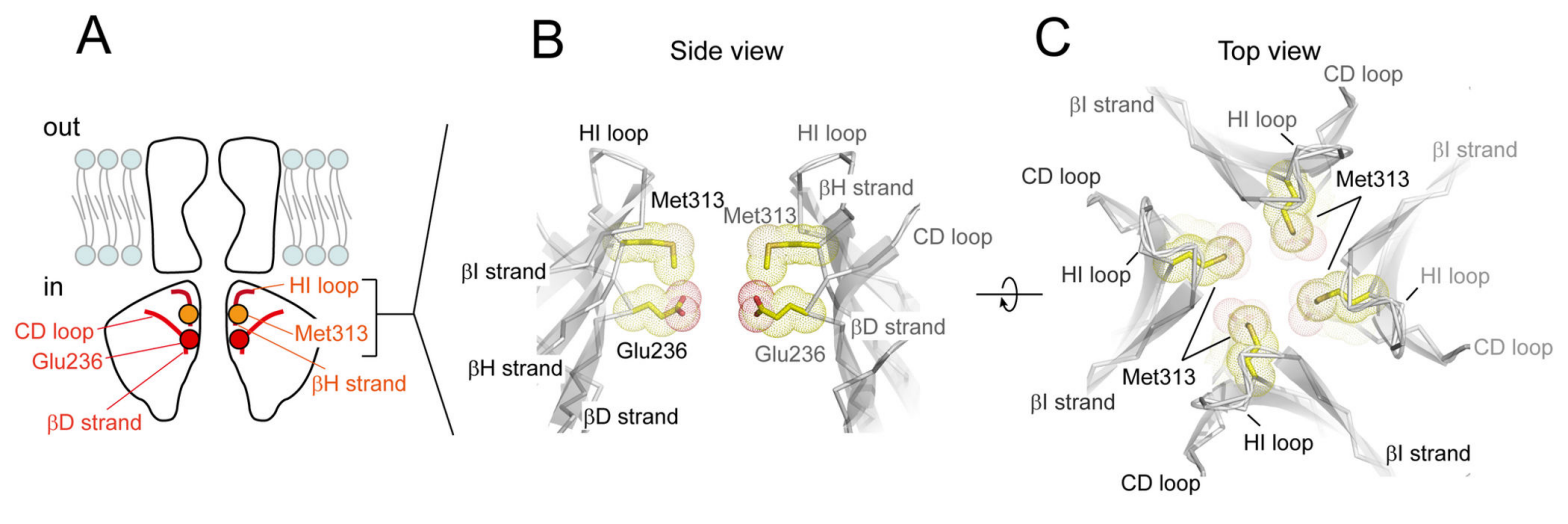
Molecular architecture of Kir3.2. *A*. Domain topology of Kir3.2. The Kir channel comprises the transmembrane domain (TMD) and cytoplasmic domain (CPD). The ion permeation pathway is located at the center of the tetrameric assembly. Glu236 and Met313 are present on adjacent βD and βH strands, respectively. *B*, *C*. Enlarged side (*B*) and top (*C*) views of the region of the CPD of Kir3.2 near the TMD. The ribbon trace represents the Cα backbone of the Kir3.2 model, and Glu236 and Met313 are shown as sticks and spheres. For clarity, the front and rear subunits in the assembly are omitted from the side view (*B*).

G protein-gated Kir channels participate in the formation of slow inhibitory postsynaptic potentials in neurons and bradycardia in the heart [1]. Four members of this channel subfamily, namely Kir3.1–Kir3.4, assemble to form a functional unit comprising a transmembrane domain (TMD) and cytoplasmic domain (CPD; Figure 1A) [2]. The direct association of the G protein βγ subunits (Gβγ) with the CPD of the channels physiologically triggers channel activation [3–6]. In addition, phosphatidylinositol 4,5-bisphosphate (PIP_2_), which is required for the activation of all Kir channels, binds the C-linker, which connects the 2 domains [7,8], and residues in the CPD [9]. The TMD primarily functions as a gate, while the CPD receives various stimuli to control the gate. Therefore, clarifying the structural elements involved in conformational changes within the CPD can help elucidate the mechanisms that control Kir channel gating.

 Crystallographic analysis suggested that dilation of the cytoplasmic pore is one of the macroscopic conformational changes in the CPD of mammalian Kir channels [8]. This is consistent with our previous study concerning the mode of binding of cations to an ion conduction pore in the CPD of Kir3.2. In particular, we determined that the pore is required to expand its inner diameter to lower the strength of the electrostatic field potential in order to facilitate ion diffusion [10]. Because cytoplasmic pore dilation also occurs in bacterial Kir channel homologs [11–14], the change in pore diameter at the CPD is assumed to be fundamental to Kir channel activity.

 At the domain interface between the TMD and CPD, a membrane-facing loop between the βH and βI strands (G loop) located at the top of the cytoplasmic pore is expected to participate in allosteric domain coupling [15], and function as a cytoplasmic gate for ion conduction [16,17]. However, the loop is variably positioned with an adjacent loop between the βC and βD strands (CD loop) identified in the crystal structures in the presence [8,18–20] and absence of the TMD [16,21]. Therefore, the role played by the rearrangement of structural elements within the CPD in the control of Kir channel activity remains unknown.

 In this study, we focused on 2 amino acids that face the cytoplasmic pore when Kir3.2 is closed: Glu236 and Met313 (Figure 1). These amino acids are spatially adjacent to each other, but present on neighboring β-strands [10]. We hypothesized that, if the expansion of the cytoplasmic pore is associated with G protein-dependent activation, the side chains of Glu236 and Met313 may shift position and contact their surroundings upon the transition from the closed state to the open state. Therefore, we analyzed the properties of Kir3.2 mutated at Glu236 and Met313 to gain insights into the role of structural elements within the CPD in the control of pore dilation and the relationship between structural rearrangements and the control of channel activation.

## Materials and Methods

### Two-electrode voltage clamp experiment

The mouse Kir3.2d isoform was subjected to functional analyses [22]. The amino acid numbers correspond to the longest mouse Kir3.2 isoform. Point mutations were introduced using the QuikChange site-directed mutagenesis kit (Stratagene, La Jolla, CA), and the presence of the mutations was confirmed by direct DNA sequencing. The care of *Xenopus laevis* was in accordance with the guideline of the Institute of Experimental Animal Sciences, Osaka University. The protocol was approved by the Animal Experiment Committee of Osaka University. The frogs were deeply anesthetized by immersing in 0.2% tricaine methanesulfonate for 20 min at 18°C. A portion of an ovary lobe was surgically removed and incubated with collagenase to remove follicular cells. The oocytes were injected with the cRNAs of Kir3.2 and porcine m_2_-muscarinic receptor (m_2_R), and maintained in modified Barth’s solution at 18°C with daily solution changes. Electrophysiological studies were undertaken 48–72 h later at room temperature (20–25°C). Two-electrode voltage-clamp recordings from oocytes were performed using a GeneClamp 500B amplifier (Axon Instruments, Sunnyvale, CA). The pipettes had a resistance of 0.5–1.5 MΩ when filled with 3 M KCl. The bath solution contained 40 mM KCl, 50 mM NaCl, 3 mM MgCl_2_, 0.15 mM niflumic acid, and 5 mM HEPES-KOH (pH 7.4). The currents were recorded in the absence or presence of various concentrations of acetylcholine (ACh). Ba^2+^ (3 mM) was added at the end of the recording to estimate the endogenous leak current. The data are expressed as mean values ± standard error of the mean. For the quantitative estimation of ACh-dependent current responses of the wild-type (WT) and mutant channels, the current amplitudes at the end of test pulses at each ACh concentration ([ACh]) were fitted using the following equation:

I=min+[(max−min)/(1+(EC50/[ACh])^nH)]

where *I* is the current amplitude measured at the [ACh], *n*_H_ is the Hill coefficient, and EC_50_ is the concentration that induces 50% of the maximal response. Min and max correspond to the minimum and maximal response, respectively; the min corresponds to zero, except in the cases of M313D and M313R. The values of *n*_H_ and EC_50_ were calculated using a non-linear least-squares best fit (SigmaPlot 9; Systat Software). Statistical analyses were performed by one-way ANOVA with post-hoc tests.

### Patch-clamp analysis

The Kir3.2d and m_2_R subcloned into pcDNA3 (Invitrogen) were transfected into HEK293T cells using the Lipofectamine Plus reagent (Invitrogen, Carlsbad, CA). Single-channel activity was recorded in a cell-attached configuration at a holding potential of -100 mV using a patch-clamp amplifier (Axopatch 200B; Axon Instruments). The current traces were low-pass filtered at 1 kHz and digitized at 10 kHz. The glass pipettes had resistances of 2.5–3.5 MΩ when filled with the pipette solution, which contained 135 mM KCl, 1 mM CaCl_2_, 1.6 mM MgCl_2_, 5 mM HEPES, and 5 μM ACh; the bath solution was a Tyrode solution. A 50% crossing criterion for the current amplitude was used to judge the single-channel activity.

 Inside-out patch clamp experiments were performed by the excision of patch membranes in the cell-attached configuration. The bath solution consisted of 135 mM KCl, 5 mM EGTA, 2 mM MgCl_2_ and 5 mM HEPES. The holding potential was -100 mV. We perfused ATP at the intracellular side to produce PIP_2_ at the inner leaflet of the plasma membrane, GTP (3 μM) to produce Gβγ mildly and GTPγS (10 μM) to generate Gβγ maximally [29]. The pH of all solutions was adjusted to 7.35 with KOH prior to the experiments. All experiments were conducted at ambient temperature (20–25°C).

### X-ray diffraction measurements and structure analyses

The construct encoding the CPD of Kir3.2 [23] was mutated to express an arginine at position 236. The purified protein was subjected to crystallization. Crystals were grown in sitting drops by mixing 2 μL of the protein solution with 2 μL of the reservoir solution containing 15% (v/v) ethanol, 0.1 M HEPES-NaOH (pH 7.5), and 0.2 M MgCl_2_. The crystals were cryoprotected by increasing the concentration of glycerol to 25%, mounted on nylon pins, and directly immersed in liquefied nitrogen. All procedures were performed at 4°C.

 The data were collected at the BL44XU beam line at the SPring-8 synchrotron radiation facility (Hyogo Prefecture, Japan), indexed and scaled using the HKL2000 suite [24]. The initial phase was determined by molecular replacement using the coordinates of the CPD of Kir3.2 (PDB code: 3AGW) [25]. The model was refined using programs in the CCP4i suite [26] and the graphics program Coot [27]. The statistics of the data collection and crystallographic analyses are presented in Table 1. Illustrations of the structures were prepared using PyMOL (DeLano Scientific LLC). The atomic coordinates and structural factors of the crystal structure of the CPD of the mouse Kir3.2 E236R mutant have been deposited in the Protein Data Bank under the accession code 3VSQ.

**Table 1:** Summary of the crystallographic analysis of the cytoplasmic domain of the Kir3.2 E236R mutant.

**Data collection**		
Space group		P42_1_2
Cell dimensions	*a*, *b*, *c* (Å)	85.86, 85.86, 73.05
	α, β, γ (°)	90, 90, 90
Resolution (Å)		50-2.0 (2.07-2.00) ^a^
*R*_sym_		0.125 (0.801) ^a^
*I/σI*		673.6 (26.4) ^a^
Completeness (%)		99.7 (100) ^a^
Redundancy		27.3 (27.2) ^a^
**Refinement**		
Resolution (Å)		30-2.0
No. reflections		17,846
*R*_work_/*R*_free_		21.6/26.0
No. atoms	Protein/Ligand (ions)/water	1595/9/84
*B* factors	Protein (main chain)	41.7
	Protein (side chains)/ligands/ions/waters	43.3/68.4/79.5/45.7
r.m.s.d. ^b^	Bond lengths (Å)	0.009
	Bond angles (°)	1.06
Ramachandran analysis (*%*)	Core, allowed, generously allowed, disallowed	88.6/11.4/0.0/0.0

^a^ Values in parentheses are for the highest-resolution shell.

^b^ r.m.s.d., root mean square deviation.

### Western blot analysis

Kir3.2 WT and mutants were expressed in HEK293T cells. The lysate obtained from cells grown in a well of 6-well plates were subjected to SDS-PAGE and blotted on a polyvinylidene difluoride membrane. The expressed channels were detected using the anti-Kir3.2 antibody (aG2A-5) [28].

## Results

In the closed state of Kir3.2, the side chains of Glu236 and Met313 are exposed to the cytoplasmic pore (Figures 1B and 1C) [8,10,23]. These amino acid residues are spatially adjacent to each other, but belong to neighboring β strands (βD and βH strands). We hypothesized that, if the expansion of the pore in the CPD is essential for channel activation, mutations at these residues may affect the property of receptor-dependent activation; even though they are not directly involved. Therefore, we tested m_2_R-dependent channel activation using proteins containing mutations at Glu236 (glycine, alanine, serine, aspartate, asparagine, valine, glutamine, and arginine) or Met313 (glycine, alanine, serine, threonine, aspartate, valine, glutamine, and arginine). In addition, we measured their current amplitudes at various membrane potentials to obtain their conductance-voltage (G-V) relationships.

### Effects of mutations at Glu236 and Met313 on m_2_R-dependent activation

**Figure 2:**
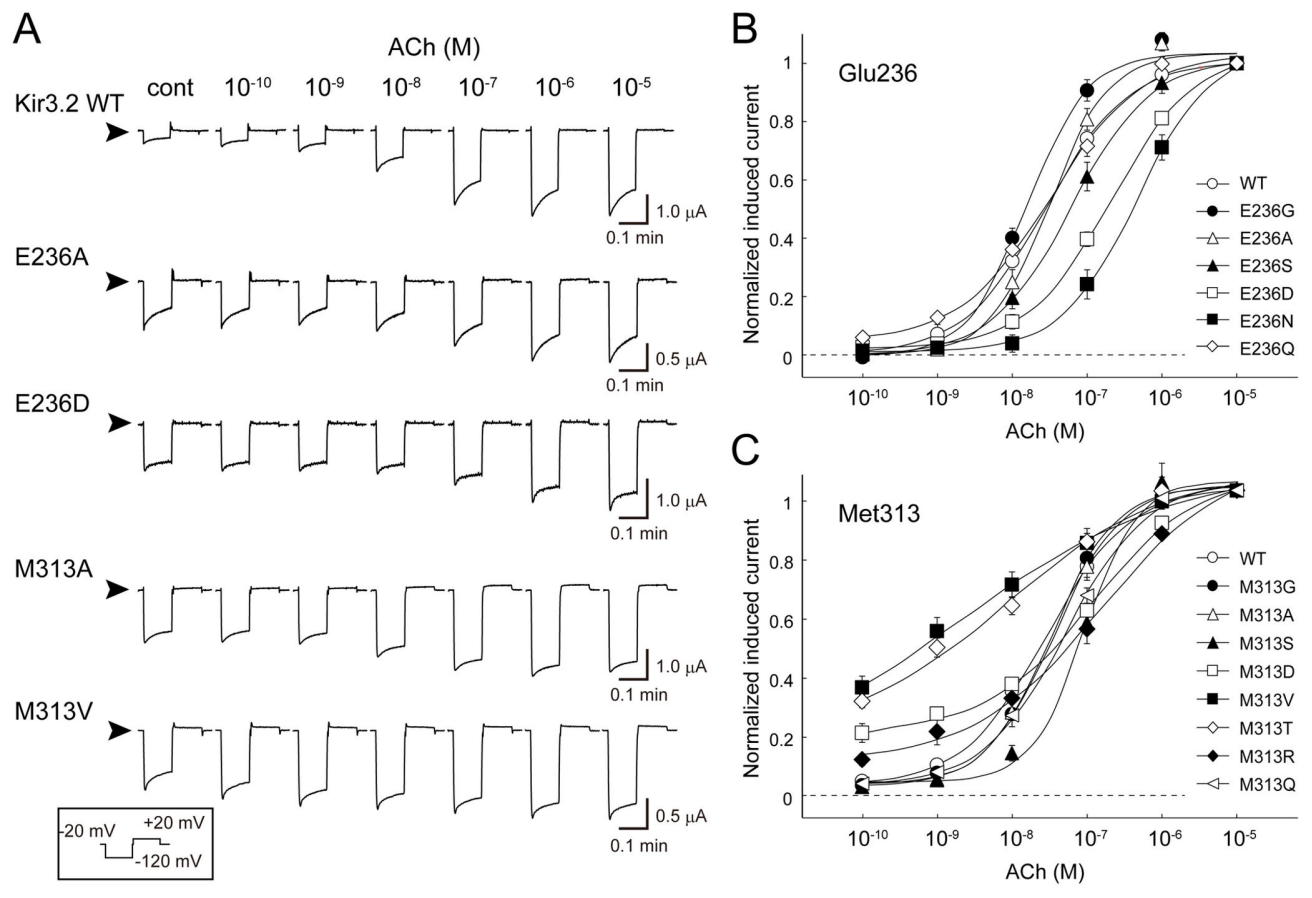
Effects of mutations at Glu236 and Met313 on m_2_R-dependent activation. *A*. Typical current traces of Kir3.2 wild-type (WT) and mutant channels recorded in the presence of various concentrations of ACh. The current responses were elicited by a test pulse at −120 mV for 0.6 s followed by a voltage step to +40 mV for 0.6 s at an interval of 0.1 s at −20 mV. This sequence was repeated every 3 s. The current in the presence of 3 mM Ba^2+^ was subtracted from each trace. The arrowheads indicate the zero current levels. *B*, *C*. Current response of Kir3.2 WT and mutant channels. ACh-induced K^+^ currents recorded at the end of the test pulse to −120 mV were normalized to that measured in the presence of 10 μM ACh. Error bars indicate the standard error of the mean. The current responses of the WT and mutants were fit using the Hill equation as described in the Materials and Methods section.

We measured K^+^ currents from *Xenopus* oocytes injected with the cRNAs of m_2_R and either WT [22] or mutant Kir3.2. Most mutants, except E236V, exhibited Ba^2+^-sensitive K^+^ currents (Figure S1 in File S1). The Ba^2+^-sensitive current responses of the WT and 4 typical mutants (E236A, E236D, M313A, and M313V), induced by various concentrations of ACh, are shown in Figure 2A. The ACh-induced current was rapidly activated and slowly decreased during hyperpolarizing voltage pulses. The ACh-induced current amplitudes at the end of the test pulse at −120 mV were normalized to that recorded in the presence of 10 μM ACh (Figures 2B and 2C). The parameters of ACh-dependent activation are summarized in Table 2. ACh activated the WT channel in a concentration-dependent manner with a Hill coefficient of 0.83 ± 0.06 and a 50% effective concentration (EC_50_) of 35 ± 8 nM (n = 12). When Glu236 was substituted with small amino acids such as glycine (E236G) and alanine (E236A), the EC_50_ values (36 ± 18 nM [n = 6] and 34 ± 5 nM [n = 11], respectively) were comparable to that of the WT. The introduction of serine (E236S), aspartate (E236D), and asparagine (E236N) increased the EC_50_ to 82 ± 17 nM (n = 9), 220 ± 30 nM (n = 9), and 430 ± 80 nM (n = 7), respectively, without significantly affecting the Hill coefficient. On the other hand, substitution to glutamine (E236Q) did not affect the EC_50_ or Hill coefficient (35 ± 6 nM and 0.75 ± 0.02, respectively; n = 10). Because the E236D and E236N mutants, but not the E236Q mutant, exhibited an obvious shift in the G-V curve to the left (Figure S2 and Table S1 in File S1), we concluded that medium-sized and non-charged amino acids efficiently prevented activation. These results suggest that Glu236 changes its position upon G protein-triggered transition from the closed state to open state, and the side chain of Glu236 associates with its surroundings.

**Table 2:** Measurement of receptor-dependent activation of Kir3.2 WT and mutants.

	**Hill equation**					**cRNA**
**Construct**	**EC_50_ (nM)**	**Hill coefficient**	***n***	**I_basal_/I_total_^a^**	***n***	**(ng/oocyte)**
WT	35 ± 8	0.83 ± 0.06	12	0.26	4	0.5
E236G	36 ± 18	1.10 ± 0.04	6	0.67^*^	4	0.025
E236A	34 ± 5	1.12 ± 0.04^*^	11	0.51^*^	22	0.025
E236S	82 ± 17	0.86 ± 0.07	9	0.50	16	0.5
E236D	220 ± 30^*^	0.74 ± 0.07	9	0.60^*^	19	0.5
E236N	430 ± 80^*^	0.82 ± 0.08	7	0.67^*^	10	25
E236V	N.A.	N.A.		N.A.		
E236Q	35 ± 6	0.75 ± 0.02	10	0.38	6	0.5
E236R	N.A.	N.A.		1.0	7	25
M313G	33 ± 2	0.98 ± 0.07	5	0.69^*^	6	0.025
M313A	28 ± 5	1.05 ± 0.08	5	0.57^*^	8	0.025
M313S	78 ± 14	1.04 ± 0.05	7	0.47	7	0.025
M313V	2.9 ± 1.3	0.36 ± 0.03^*^	6	0.78^*^	25	5
M313T	6.6 ± 2.0	0.38 ± 0.03^*^	7	0.86^*^	21	2.5
M313D	130 ± 10^*^	0.73 ± 0.06	10	0.59	5	2.5
M313Q	50 ± 5	0.81 ± 0.04	7	0.42	13	2.5
M313R	110 ± 40	0.52 ± 0.08	7	0.66^*^	10	5

*n*, number of observations; N.A., not available; *p < 0.003.

^a^ The value obtained by dividing the basal current amplitude of the Kir3.2 mutants by total current amplitude only from oocytes having a total current less than 2.5 μA.

Next, we compared the receptor-dependent activation of Kir3.2 with mutations at Met313 (Figure 2C and Figure S1 in File S1). When Met313 was substituted with glycine or alanine, the dose-response curves were similar to that of the WT; the EC_50_ and Hill coefficients were 33 ± 2 nM and 0.98 ± 0.07 for M313G (n = 5), and 28 ± 5 nM and 1.05 ± 0.08 for M313A (n = 5), respectively. The introduction of serine (M313S) slightly shifted the curve to the right (EC_50_, 78 ± 14 nM; n = 7). Mutation of Met313 to a branched residue (valine or threonine) significantly reduced the Hill coefficient (0.36 ± 0.03 for M313V [n = 6] and 0.38 ± 0.03 for M313T [n = 5]). The introduction of charged residues (M313D and M313R) resulted in shallow-sloped activation curves with high EC_50_ values. In contrast, substitution with glutamine (M313Q) did not significantly affect receptor-dependent channel activation. These results indicate that mutations at position 313 mainly affect the slope of the activation curve. The m_2_R and G proteins mediate the ACh-dependent activation of the channels. This sequential reaction is thought to render the Hill coefficients derived from receptor-stimulation smaller than those obtained by stimulation with Gβγ or GTP [29–31]. Because the difference in the current responses of mutants appears to stem from the mutants’ own properties, Met313 may shift its position upon gating and associate with its surroundings.

### Effects of mutations at position 313 of Kir3.2

**Figure 3:**
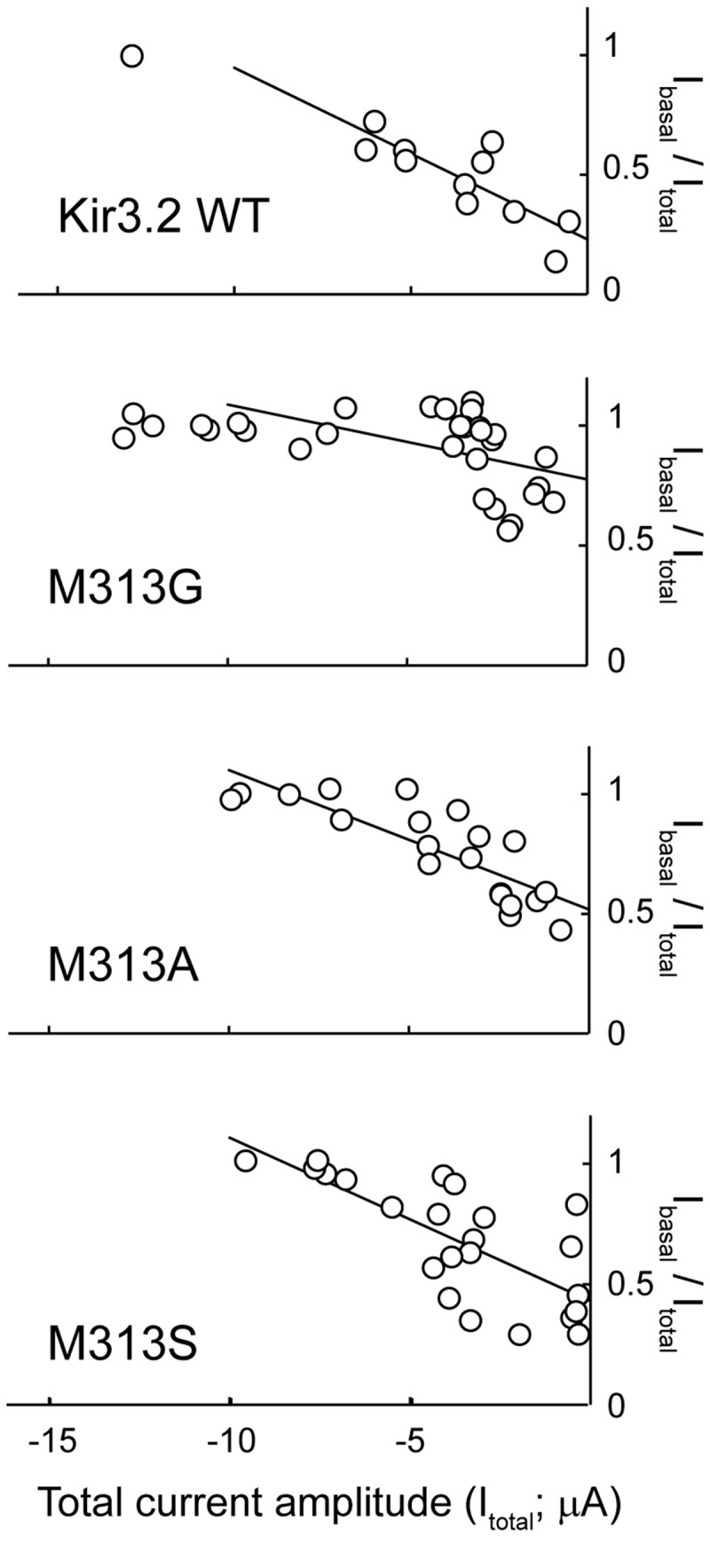
Effects of mutations at position 313 of Kir3.2 on basal activity. Comparison of the ratios of the basal current of Kir3.2 WT and mutant channels. The current amplitudes of Kir3.2 WT and mutant channels in the absence of ACh (I_basal_) were divided by those in the presence of 10 μM ACh (I_total_) in every oocyte. The I_basal_/I_total_ ratio was plotted against the amplitude of I_total_ for the WT and each mutant. The plot shows a linear relationship when the I_total_ is less than 10 μA.

**Figure 4:**
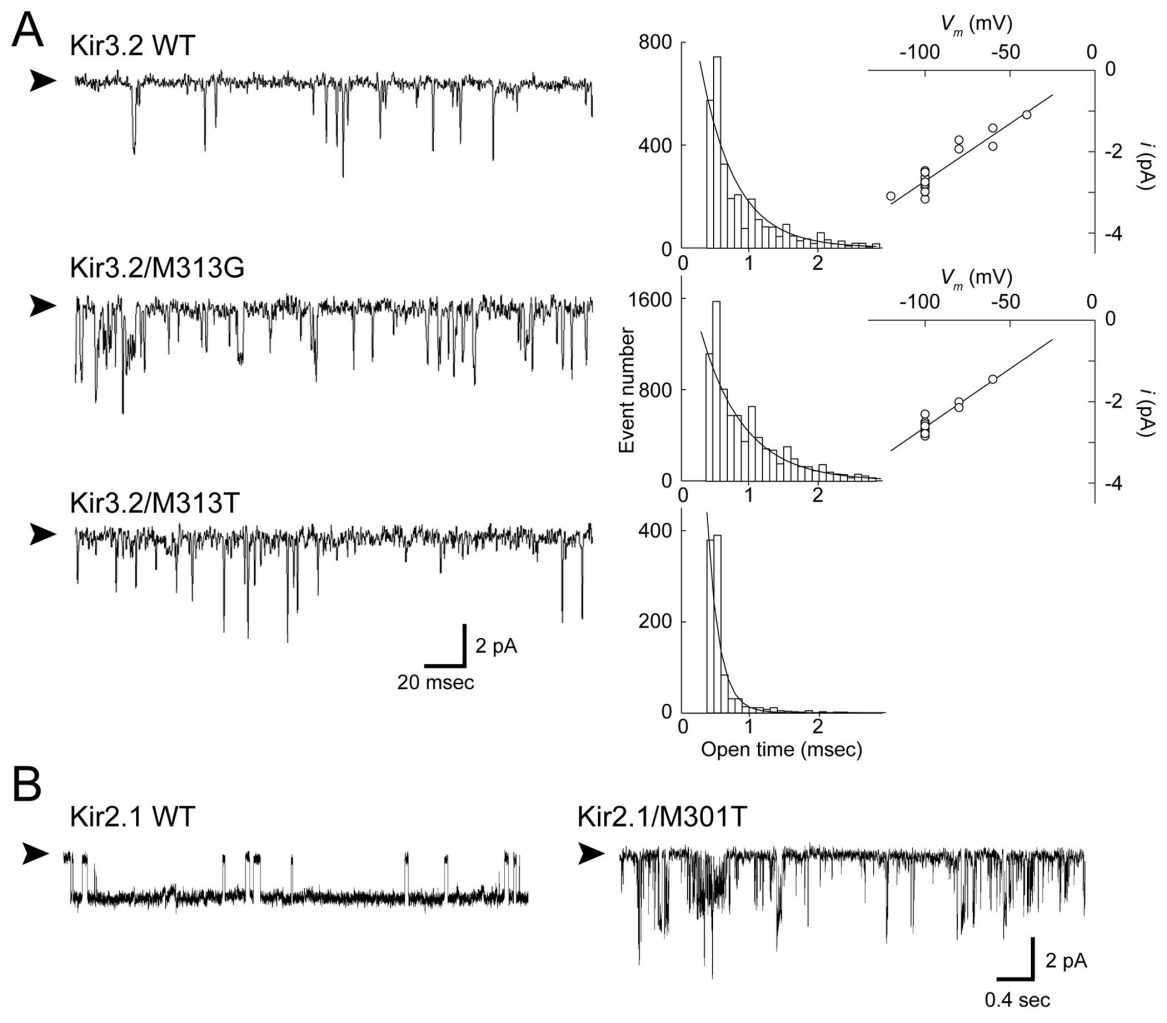
Effects of mutations at position 313 of Kir3.2 on single-channel currents. *A*. Single-channel recording of the indicated channel at −100 mV. The patch recording in the cell-attached configuration was obtained from HEK293T cells expressing Kir3.2 WT, M313G, and M313T. The holding potential was −100 mV. Open dwell time histograms for each channel are shown at the right side of the trace and are fit with a single exponential function. The single-channel current amplitudes of the WT and M313G mutant are plotted against the membrane potentials. *B*. Effects of substitutions of threonine 301 in Kir2.1. Met301 in Kir2.1 is equivalent to Met313 in Kir3.2. The substitution of threonine at Met301 in Kir2.1 resulted in a spiky opening with variable conductance. Arrowheads indicate the zero current level.

**Figure 5:**
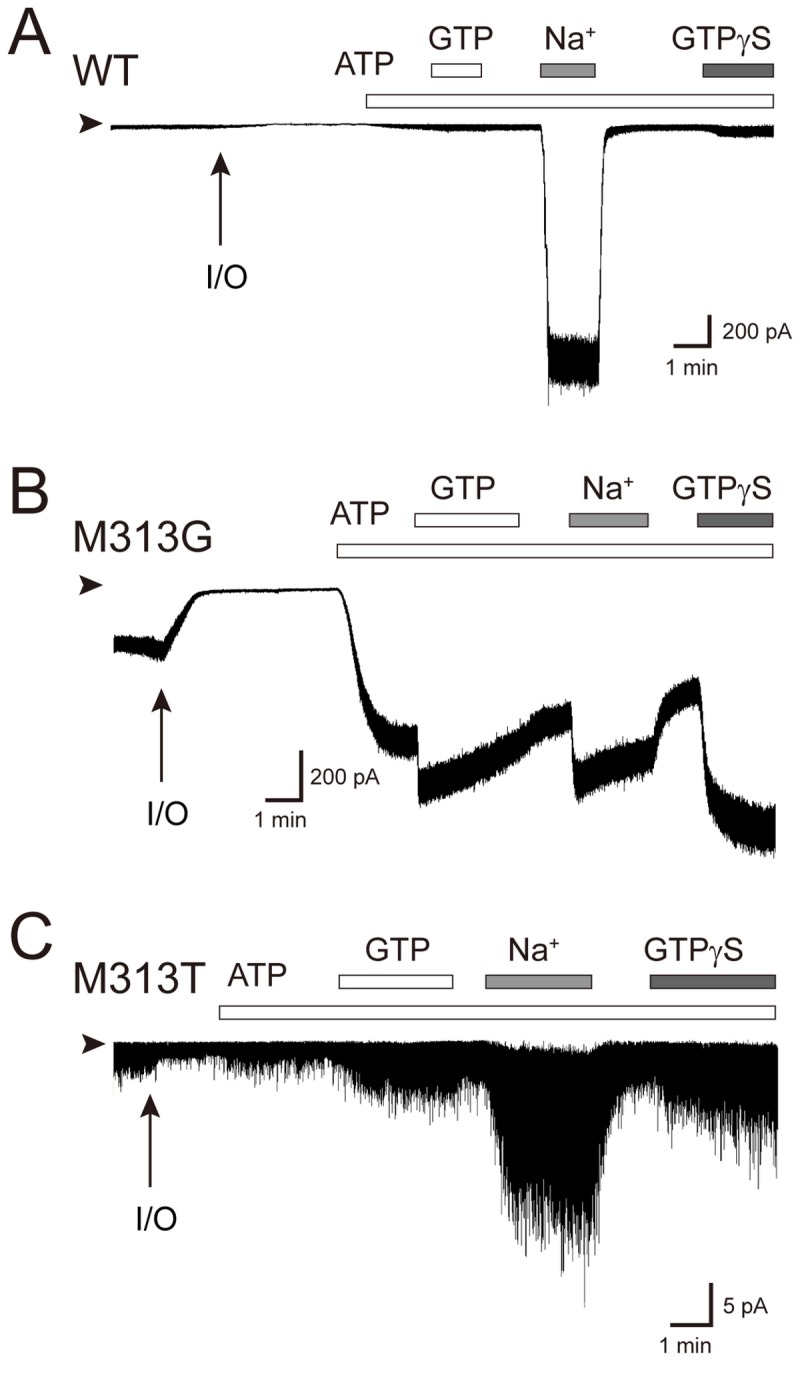
Inside-out patch recordings of Kir3.2 WT and mutant channels. Inside-out membrane patches were obtained from HEK293T cells expressing either Kir3.2 WT (*A*), M313G (*B*) or M313T mutant (*C*) channels. Experiments were conducted in symmetric 135 mM K^+^ solutions with a holding potential of -100 mV. Perfusion of ATP was for the generation of PIP_2_ at the inner leaflet of the excised patch membranes. The protocol used for perfusion of substances to the intracellular side of the patch membrane is indicated by bars above the current traces.

When the expression level of the G protein-gated Kir channels was increased, the cells exhibited the K^+^ current, even in the absence of receptor stimulation [32,33]. When this basal current amplitude (I_basal_) was divided by that in the presence of 10 µM ACh (I_total_) in every oocyte, the plot of the I_basal_/I_total_ fraction against the I_total_ showed a linear relationship in the WT and mutant channels when the I_total_ was less than 10 µA (Figure 3). Assessment of the basal current fraction of the Kir3.2 WT and mutants only from oocytes with a total current of less than 2.5 μA revealed that many mutants had a higher fraction of I_basal_ than the WT (Table 2). This suggests that the presence of Glu236 and Met313 plays a role in stabilizing the closed state, and supports the idea that the conformation of the CPD of Kir3.2 changes around the cytoplasmic pore [10]. However, because the EC_50_, Hill coefficient and I_basal_/I_total_ values of the mutants varied depending on the mutated residues and introduced residues, it is not necessarily appropriate to suggest that the effects of the mutations could simply be compared. Nevertheless, it appears that the I_basal_/I_total_ values of some of the Met313 mutants are worthy of note. These mutants contained a small side chain (M313G, M313A, and M313S), exhibited EC_50_ and Hill coefficient values comparable to those of the WT (Figure 2 and Table 2), and G-V relationship comparable to that of the WT (Figure S2 in File S1). However, reductions in the volume of the side chain dramatically increased the I_basal_/I_total_ ratio (Figure 3). To reveal the mechanism underlying this phenomenon, we measured the single-channel activity of the mutants at position 313 in Kir3.2. As shown in Figure 4A, in the cell-attached patch configuration, Kir3.2 WT exhibited a spiky opening with a mean open time of 0.36 ± 0.02 ms (n = 18) and a single-channel conductance of 28 pS [22]. The M313G mutant exhibited a prolonged mean open time of 0.82 ± 0.10 ms (n = 8, *p* = 0.002) with a single-channel conductance (29 pS), similar to that of the WT. This suggests that the mutation leads to the preferential existence of the open state; moreover, this property could account for the increase in the basal current level. The increased I_basal_ may be attributed to endogenous Gβγ trafficking with newly synthesized channels to the plasma membrane [4,32,33]. The mutations in the TMD are also reported to increase the I_basal_ [34]. To identify which mechanism, Gβγ-dependent or Gβγ-independent, contributes to the increased I_basal_ in M313G, we expressed the Kir3.2 WT and mutant channels in HEK293T cells and measured their activity in the inside-out patch configuration (Figure 5). After excising the patches, ATP was perfused at 2 mM to produce PIP_2_ at the inner leaflet of the patch membranes. The WT was weakly activated by ATP perfusion and its activity was 15 ± 4% of that evoked by 10 μM GTPγS (n = 7). However, M313G was remarkably stimulated, by 59 ± 6% (n = 5). Taken together, the increase in the affinity to PIP_2_ is likely to be responsible for the prolonged mean open time and the increased I_basal_ of M313G.

 In contrast, the M313T mutant, which exhibited a reduced Hill coefficient (Figure 2), showed a mean open time (0.16 ± 0.02 ms; n = 6) that was much shorter than that of the WT (Figure 4A). The protein expression level of M313T was comparable to that of the WT (Figure S4 in File S1). Thus, the shortened mean open time of the single channel (Figure 4) was consistent with the observation of low current expression in HEK cells (Figure 5) and the requirement for 5-fold more cRNA to be injected into the *Xenopus* oocyte to record a current amplitude comparable to that of the WT (Table 2). In contrast to M313G, M313T was less sensitive to ATP, but it was activated by GTP and exhibited about 11 ± 2% of the response evoked by GTPγS (n = 5), which was higher than the WT (4 ± 2% [n = 5]). It was difficult to identify a single-channel conductance in M313T (Figure 4A). Because its brief dwell time in the open state might prevent the estimation of the single-channel property, we introduced threonine at position 301 of Kir2.1, which is equivalent to Met313 of Kir3.2, and measured its single-channel activity (Figure 4B). The mutation obviously conferred a shorter open time and unstable conduction. It was also difficult to estimate the single-channel conductance of the Kir2.1 M301T mutant. These results suggest that, even though Met313 is situated in the CPD of Kir3.2, its side-chain effectively affects the gate. Furthermore, the comparable phenotype associated with these mutants implies that, even though Kir3.2 is sensitive, whereas Kir2.1 is insensitive to Gβγ, they share a similar conformational change around the methionine on their βH strand during gating. Moreover, although M313G and M313T mutants retained the sensitivity to channel activators, they significantly reduce the sensitivity to Na^+^. These observations suggest that conformational change around Met313 participates in the Na^+^-dependent activation.

### Introduction of a positive charge at position 236 in Kir3.2

**Figure 6:**
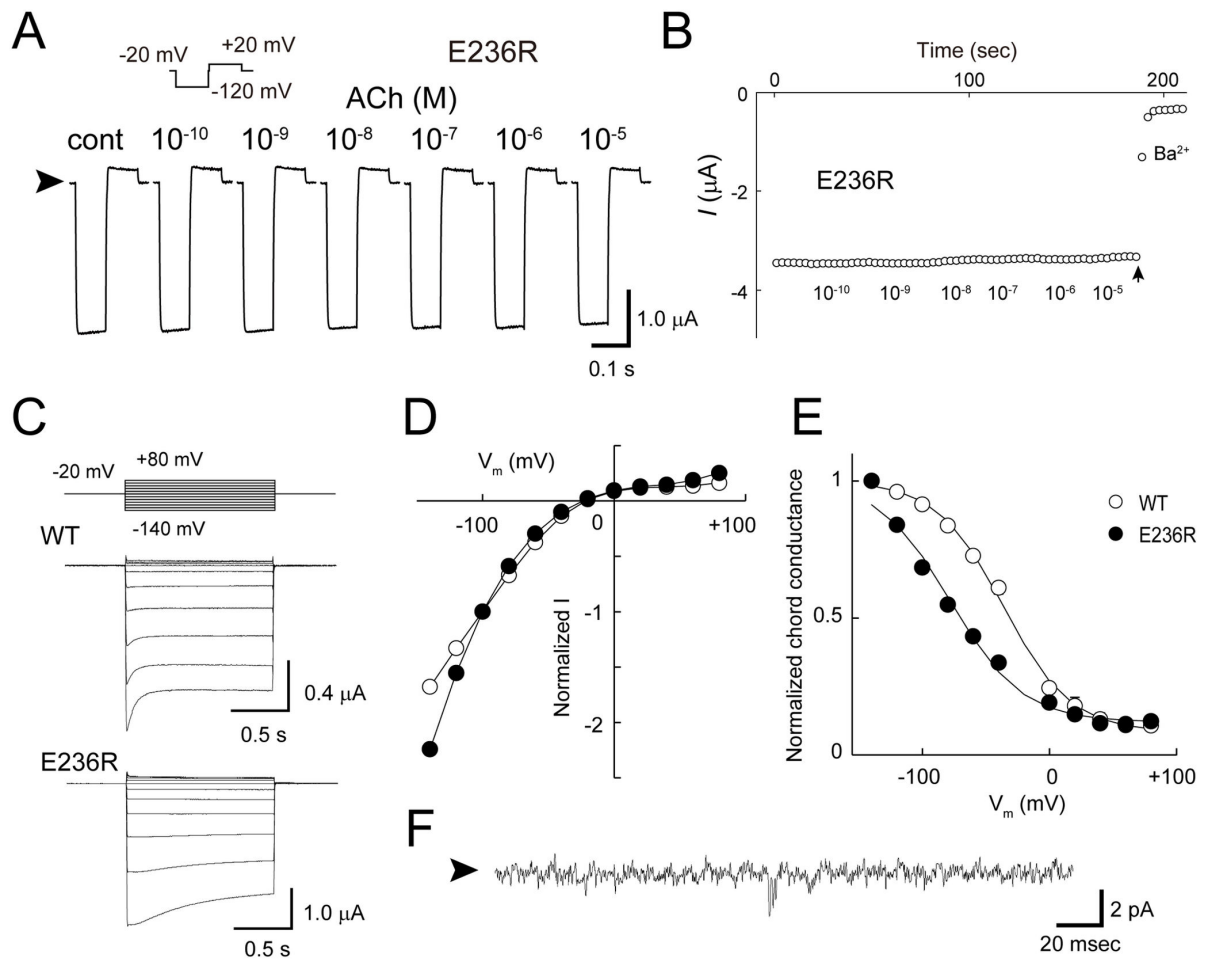
Introduction of reverse charge at position 236 of Kir3.2. *A*. Current traces of the Kir3.2 E236R mutant. Typical current traces of the E236R mutant recorded in the presence of various concentrations of ACh are shown. The Ba^2+^-insensitive current component was subtracted. Arrowheads indicate zero current levels. *B*. Whole-cell current amplitudes of the E236R mutant. The K^+^ currents recorded at the end of the test pulse to −120 mV show that the mutant is insensitive to m_2_R-stimulation. The arrow indicates the time at which Ba^2+^ was loaded into the bath solution. *C*. Whole-cell currents from oocytes expressing Kir3.2 WT and E236R mutant channels. Macroscopic currents were induced by voltage steps (1.2 s) from the holding potential of −20 mV to potentials from −140 to +80 mV in 20-mV increments. The Ba^2+^-sensitive currents of the WT and E236R channels in the presence of 10 μM ACh are shown. *D*. Current-voltage relationship. The current amplitudes at the end of the test pulse were normalized to that recorded at -100 mV. *E*. Conductance-voltage relationship. The voltage dependence of channel activation was determined from the relationship of chord conductance to voltage according to the following Boltzmann equation. $g/g_{\max}=g_{\min}+\left(1-g_{\min}\right)/\left(1+\exp\left[\{V-V_{1/2}\}/k\right]\right)$ where *V*_1/2_ is the half-maximal activation voltage and *k* is the slope factor: WT: V_1/2_ = −34 ± 4 mV, *k* = 27 ± 3 mV (n = 10); E236R: V_1/2_ = −78 ± 1 mV, *k* = 28 ± 1 mV (n = 11). Error bars indicate the standard error of the mean. All error bars are smaller than the symbols used. *F*. Single-channel recording of E236R. Channel currents were recorded from HEK293T cells in the cell-attached patch-clamp configuration. The arrowhead to the left of the trace represents the zero current level.

Mutational analyses suggested that receptor stimulation causes a positional shift in Glu236 (Figure 2). To further explore the structural rearrangement around Glu236, we tested the effect of a reverse charge at this position by introducing arginine (E236R). Even though its protein expression level was similar to that of the WT in HEK cells (Figure S4 in File S1), about 50-fold more E236R cRNA was required to obtain a current amplitude comparable to that of the WT (Table 2). The mutation led to a Ba^2+^-sensitive K^+^ current and rendered the mutant constitutively active (Figures 6A and 6B). The current-voltage relationship of the mutant exhibited strong inward rectification (Figures 6C and 6D). Meanwhile, the mutant exhibited a shift in the G-V relationship towards the negative direction (Figure 6E). A Kir2.1 mutant having the equivalent mutation to E236R of Kir3.2 (E224R) also conferred a rectification property similar to that of E236R on the mutant [35,36]. Next, we recorded the single-channel activity of the E236R mutant (Figure 6F). The mutant showed rare channel openings with a single-channel conductance that was much smaller than that of the WT (Figure 4A). The properties of a mutant in which lysine was substituted at Glu236 (E236K) was similar to that of the E236R mutant (Figure S3 in File S1). Thus, the introduction of positive charge at position 236 appeared to yield insensitivity to Gβγ and cause a leftward shift in the G-V relationship.

**Figure 7:**
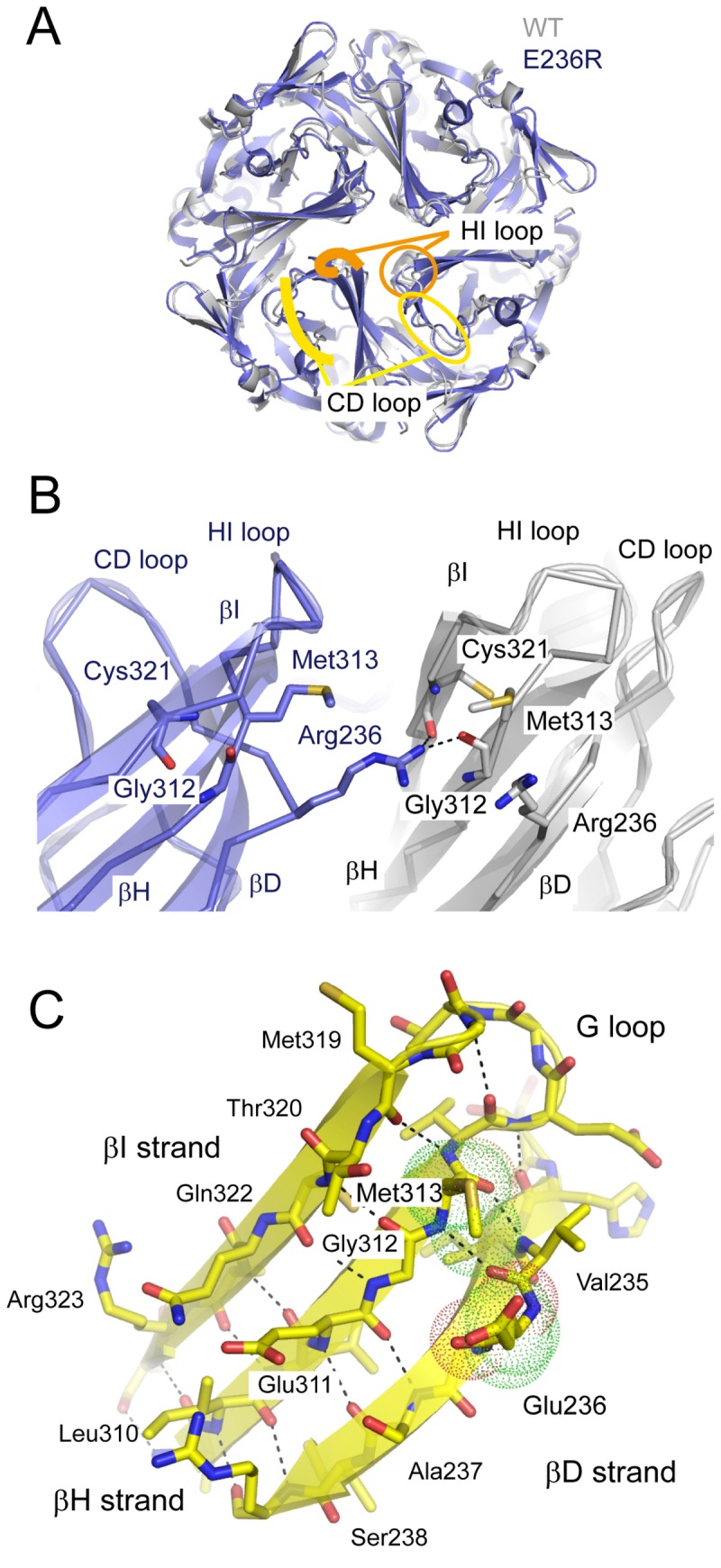
Crystal structure of the Kir3.2 E236R mutant. *A*. Comparison of the crystal structures of the cytoplasmic domains (CPDs) of WT and E236R channels. The CPD of the E236R mutant (blue) behaves as a tetramer and has a structure similar to that of the WT in a closed conformation (gray). The βC and βD strands (CD loop; yellow) and HI loop (orange) are denoted by circles in 1 subunit and traced with lines in the next subunit. *B*. Enlarged view of the subunit interface at position 236. An arginine introduced at site 236 of one subunit (blue) forms hydrogen bonds with the carbonyl oxygen atoms of Gly312 on the βH strand and Cys321 on the βI strand of the adjacent subunit (gray). The residues crucial for the interaction are shown as sticks. *C*. The β-bulge in the βD strand. The β sheet observed from the pore is shown with ribbons and sticks. The side chains of Glu236 and Met313 are shown with dots. The β-type hydrogen bonds are disconnected between Glu236 and Gly237 on the βD strand and Gly312 on βH strand. The dashed line indicates the hydrogen bond between the main chain atoms.

Because Glu236 is present in the CPD, the characteristics of the E236R mutant could stem from the properties of the CPD. Therefore, we performed crystallographic analysis using a protein construct of the CPD of Kir3.2 containing an E236R mutation [23]. The mutant protein could be purified and crystallized under conditions similar to those of the WT (Table 1) [25]. The protein existed as a tetramer in both solution and crystals (Figure 7A). The positions of the backbone Cα atoms in the E236R core structure were similar to those in the WT structure (root mean square deviation, 1.0 Å), suggesting that the mutant structure is comparable to a closed conformation [10]. It is noteworthy that the introduced arginine contacts the adjacent subunit via hydrogen bonding with the carbonyl oxygen atoms of Gly312 on the βH strand and Cys321 on the βI strand (Figure 7B). The negative electrostatic field potential at the surface of the cytoplasmic pore of Kir3.2 [10,38] may facilitate the formation of inter-subunit bonds between the positively charged side chain introduced at position 236 and backbone carbonyl oxygen atoms.

 These inter-subunit hydrogen bonds appear to exert 2 distinct effects on the mutant structure. The first is the immobilization of the βD strand. This strand connects to the CD loop, which shifts its position upon receptor stimulation [25,37]. The loss of pliability of the βD strand may prevent the G protein-dependent conformational change and render the E236R mutant insensitive to G proteins. The second is the immobilization of a pore-forming β sheet consisting of the βD, βH, and βI strands. The distance between the Cα atoms at site 236 in the diagonal subunits of the E236R structure was 20.1 Å, which is slightly wider than that in the closed state of the WT structure (16.5 Å). This feature may be insufficient to prevent K^+^ passage, even though it possesses a strong negative electrostatic potential [10,38]. Cys321 of Kir3.2 is accessible to sulfhydryl modifiers from the cytoplasmic side [37]. The position tends to adapt to the amino acid substitution and the property of the parent dominates those of the mutants (Figure S3 in File S1) [37]. On the other hand, although neither the G312A nor the G312Y mutation on the E236R mutant was functional, the corresponding mutant of Kir2.1 (G300A) retained the WT property [16]. Therefore, the introduction of arginine at position 236 may affect the rigidity of the pore-forming β sheet and constrain the full opening of the cytoplasmic pore, rather than deteriorate the domain coupling mediated by the side chains of Gly312 and Cys321. Taken together, these inter-subunit bonds may contribute to the unique conduction of the E236R mutant.

## Discussion

The Kir channel is a ligand-operated membrane protein, and its CPD receives various stimuli to control the gate at the TMD. In this study, we analyzed conformational changes around the cytoplasmic pore in the CPD of Kir3.2 by focusing on the pore-facing residues Glu236 and Met313. Even though these 2 residues are spatially adjacent to each other in the closed conformation, the effects of mutagenesis on receptor-dependent activation differed between them. In particular, the Glu236 mutants had increased EC_50_ values and the Met313 mutants had decreased Hill coefficient values (Figure 2). Furthermore, the inter-subunit connections generated in the E236R mutant appear to result in a leftward shift in the G-V relationship and G protein insensitivity (Figures 6 and 7). These results indicate that, when Kir3.2 functions, the CPD substantially alters its conformation. In particular, the region around the cytoplasmic pore is altered and the most likely change is associated with the expansion of the pore.

 In the crystal structures of Kir3.2, Glu236 protrudes into the cytoplasmic pore (Figure 1). This conformation is supported by the disruption of the β-type hydrogen bonds between the amino acids at 236 and 237 on the βD strand, and a residue at 312 on the βH strand (Figure 7C). This structural feature is known as a β-bulge [39], which is thought to confer structural flexibility to the β sheet. The β-bulge is also observed at the corresponding regions of Kir3.1 and Kir2.1 [16], and Kir3.1 exhibits significant displacement of the backbone at a residue corresponding to Glu236 of Kir3.2 and the connecting CD loop in 2 different crystal forms [16,21]. We previously reported that interference of the conformational change of the CD loop by Cd^2+^ [37] or its N-terminus [25] hampers the activation of Kir3.2. The displacement of the CD loop is expected to be coupled to PIP_2_-binding [25,40]. Therefore, the G protein might allow the channel to bind PIP_2_ and then shift the position of the βD strand and the connecting CD loop as a unit.

 The βH strand, which harbors Met313, is also expected to shift its position during gating (Figures 2, 3, 4, and 5, and Figures S1 and S2 in File S1). The βH strand associates with the βI strand by β-type hydrogen bonds and specifically contacts the arginine introduced at position 236 of the adjacent subunit in the E236R mutant (Figure 7). Thus, it could be anticipated that the βH strand forms an element with the βI strand and the structurally flexible G loop that is sandwiched by the 2 strands. The introduction of threonine at Met313 in G protein-sensitive Kir3.2 and at the corresponding residue in G protein-insensitive Kir2.1 notably resulted in the same phenotype: short open dwell time and unstable conductance at the single-channel level (Figure 4). Because the mutations at Met313 potentially disturb the conformation of the G loop, these findings are insufficient to exclude the possibility that the G loop functions as a cytoplasmic gate [16,17]. Nevertheless, these results suggest that the role of the βH strand and its surroundings in the CPD in the regulation of gating is similar in Kir channels despite the sensitivity to G proteins. The most recent crystal structure of Kir3.2 suggests that the G loop is sandwiched by 2 TM2s of adjacent subunits [20]. Therefore, it might be possible that the element accommodating the βH strand concomitantly moves with the TMD at each step of single-channel gating, even though the element is part of the CPD. 

 So far, the G protein-gated Kir channels have been proposed to exhibit rotational motion of the CPD against the TMD during gating [41]. Based on the crystal structure of mammalian Kir3.2 in complex with PIP_2_, Na^+^ and Gβγ [20], it is proposed that the interaction of Gβγ at the interface of the subunit in the CTD evokes the twist against the TMD without disturbing the conformation of the CTD. The manner of this CTD-originated rotational motion of the channel is in sharp contrast with the motion observed in KcsA, in which its TMD twists during gating by itself [42]. A ligand-induced conformational change at the ligand-binding domain is thought to mechanically force the opening of the activation gate at the TMD in ligand-operated ion channels such as ionotropic glutamate receptors [43,44] and Ca^2+^-activated K^+^ channels [45–47]. Even though the pore-forming βD and βH strands contribute to the dilation of the cytoplasmic pore, they seem not to move as a combined unit. This β-bulge-based structural feature of the βD and βH strands raises the question of whether the conformational change in the βD strand generates the tension that drives the TMD by regulating the position of the βH strand. Furthermore, how such microscopic rearrangements of these structural elements couple to the macroscopic conformational changes observed at the domain level should be clarified in the near future. Further structural data on the open channel state combined with mutational analyses are required to elucidate the functional implications of pore dilation in the CPD and the coupling between the 2 domains in the regulation of Kir channel gating.

## Supporting Information

File S1 (PDF)
