# Supplementary material for: Conformational Changes Underlying Pore Dilation in the Cytoplasmic Domain of Mammalian Inward Rectifier K+ Channels
Source: PLoS One. 2013 Nov 11;8(11):e79844. doi: 10.1371/journal.pone.0079844 (PMC3823594; doi:10.1371/journal.pone.0079844)
Supplement: File S1 — (PDF) [file pone.0079844.s001.pdf]

Supplemental information

Conformational Changes Underlying Pore Dilation in the Cytoplasmic Domain of Mammalian Inward Rectifier K^+ Channels

Atsushi Inanobe, Atsushi Nakagawa, and Yoshihisa Kurachi

Figure S1. Current responses of Kir3.2 WT and mutant channels activated by m_2R

Typical current responses of Kir3.2 WT and mutant channels were recorded by a two-electrode voltage-clamp. The membrane potential was held at −20 mV and stepped to −120 mV for 0.6 sec, followed by a voltage step to +40 mV for 0.6 sec, with an interval of 0.1 sec at −20 mV. The current amplitudes at the end of the test pulse at −120 mV obtained from oocytes were plotted. The channels were activated by increasing concentrations of ACh as indicated in each panel. The current responses at each concentration of ACh are shown in the inset of each panel. The current traces with the darkest color represent the absence of ACh and the color gradually becomes lighter with increasing concentrations of ACh. Ba^{2+} (3 mM) was also added at the end of the experiment to estimate the Ba^{2+}-sensitive current response. The introduction of valine (E236V) resulted in smaller currents despite the injection of 100-fold more cRNA than used for the WT. The horizontal and vertical bars in the insets indicate 100 msec and 1 μA, respectively.

Figure S2. Conduction profiles of Kir3.2 WT and mutant channels

A. Whole-cell currents were recorded from *Xenopus* oocytes expressing various Kir3.2 WT and mutant channels. The pairs of current traces of Kir3.2 WT and mutants (E236G, M313T and M313D) were obtained by subtraction of the Ba^{2+}-insensitive currents from the currents recorded in the presence of ACh. The bath solution contained 45 mM K^+. Voltage steps (1.2 sec in duration) from the holding potential of -20 mV to potentials between +40 mV and -140 mV, with -20 mV increments, were delivered at a rate of every 5 seconds.

B. The current-voltage relationship presents the current amplitudes at the end of each pulse for each set of oocyte injections. Since the current amplitudes varied depending on oocytes, the relative current amplitude at various membrane potentials were normalized to that recorded at -100 mV.

C. The chord conductance-voltage relationship

The voltage dependence of channel activation was determined from the relationship of chord conductance to voltage from the potassium equilibrium potential as described in the legend to Figure 6.

Figure S3. Electrophysiological property of mutants at position 236 of Kir3.2

A. Effect of the introduction of positive charge at position 236

Kir3.2 WT, E236R, and E236K mutants were expressed in *Xenopus* oocytes with m_2R. The currents recorded before (blue) and after the application of ACh (red), as well as in the presence of Ba^{2+} (black) are shown. The current amplitudes at the end of test pulse at -120 mV obtained from oocytes were plotted. The combination of test pulses is also shown above the current traces.

B. Electrophysiological properties of E236R and E236K mutants

The Ba^{2+}-sensitive macroscopic currents of the E236R and E236K mutants are shown. Their current-voltage and chord conductance-voltage relationships are also shown.

C. Effect of the second mutation on the properties of the E236R mutant

Mutants with a second mutation at position 312 were non-functional. However, the residue positioned at 321 adapted to substitution with methionine and threonine.

Figure S4. Western blot analysis of Kir3.2 WT and mutant channels

Kir3.2 and mutants (M313G, M313V and E236R) were expressed in HEK293T cells. The lysate obtained from cells grown in a well of 6-well plates were subjected to SDS-PAGE and blotted on a polyvinylidene difluoride membrane. The expressed channels were detected using the anti-Kir3.2 antibody named aG2A-5.

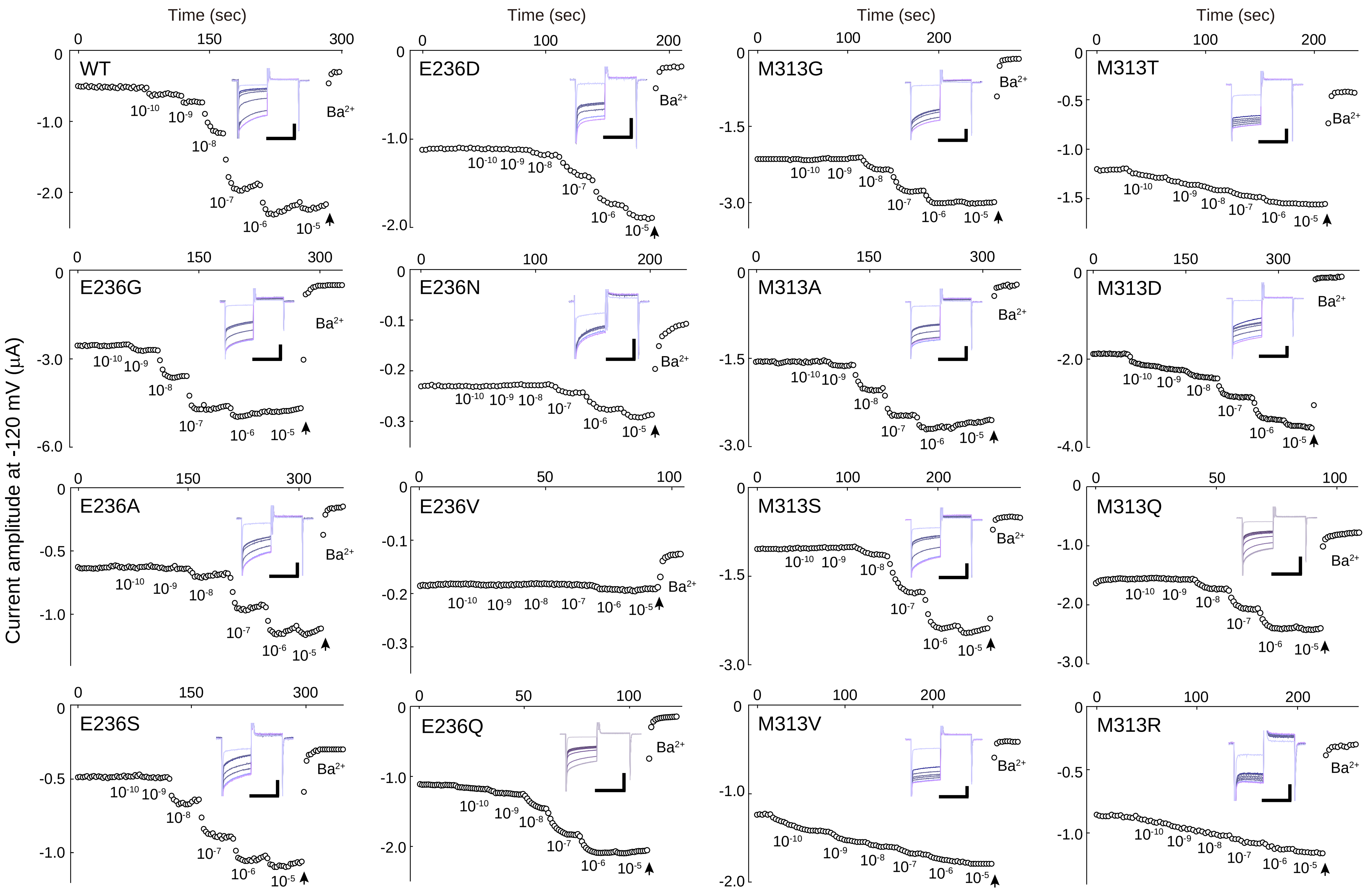

Figure S1. Inanobe et al

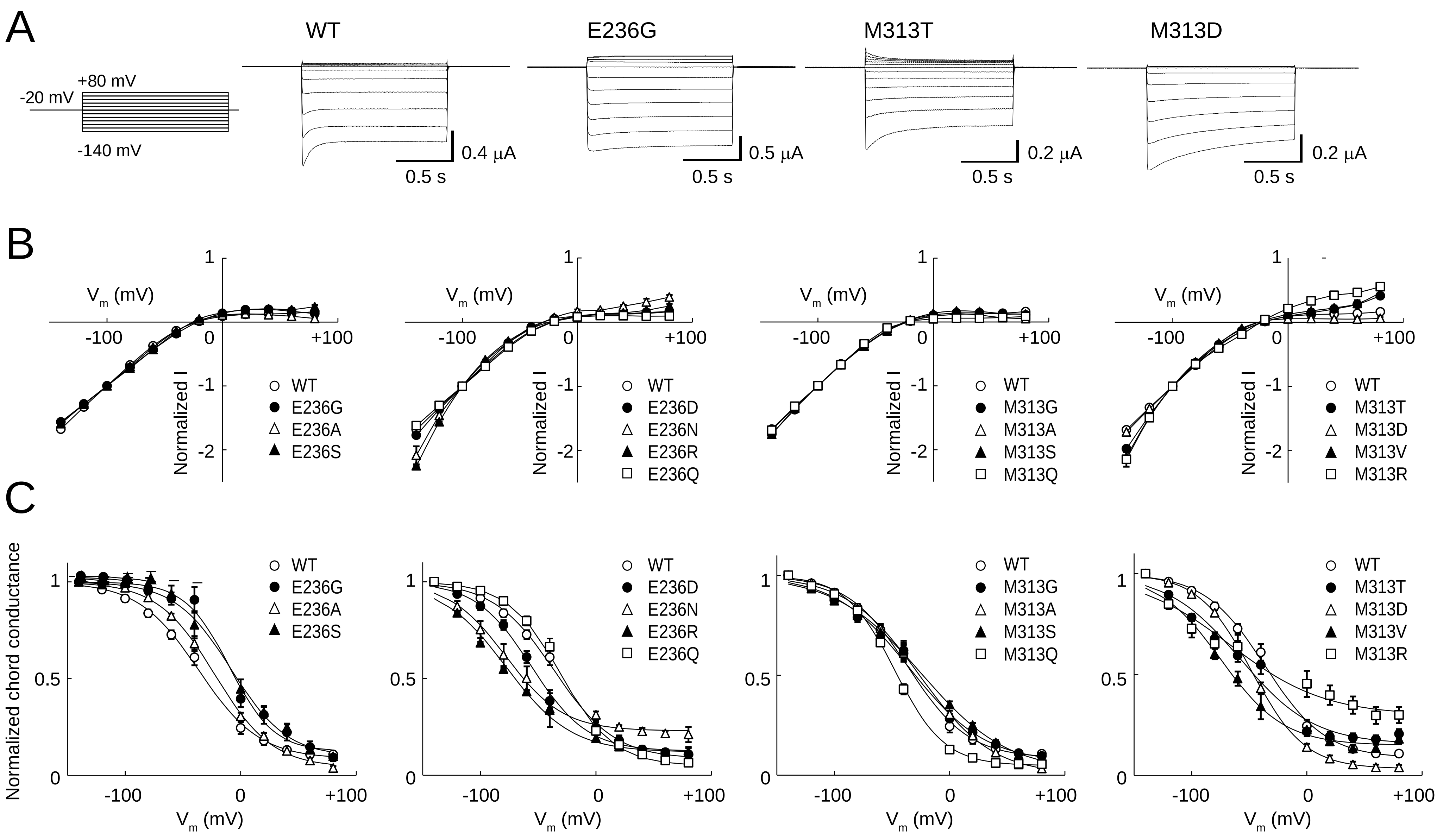

Figure S2. Inanobe et al

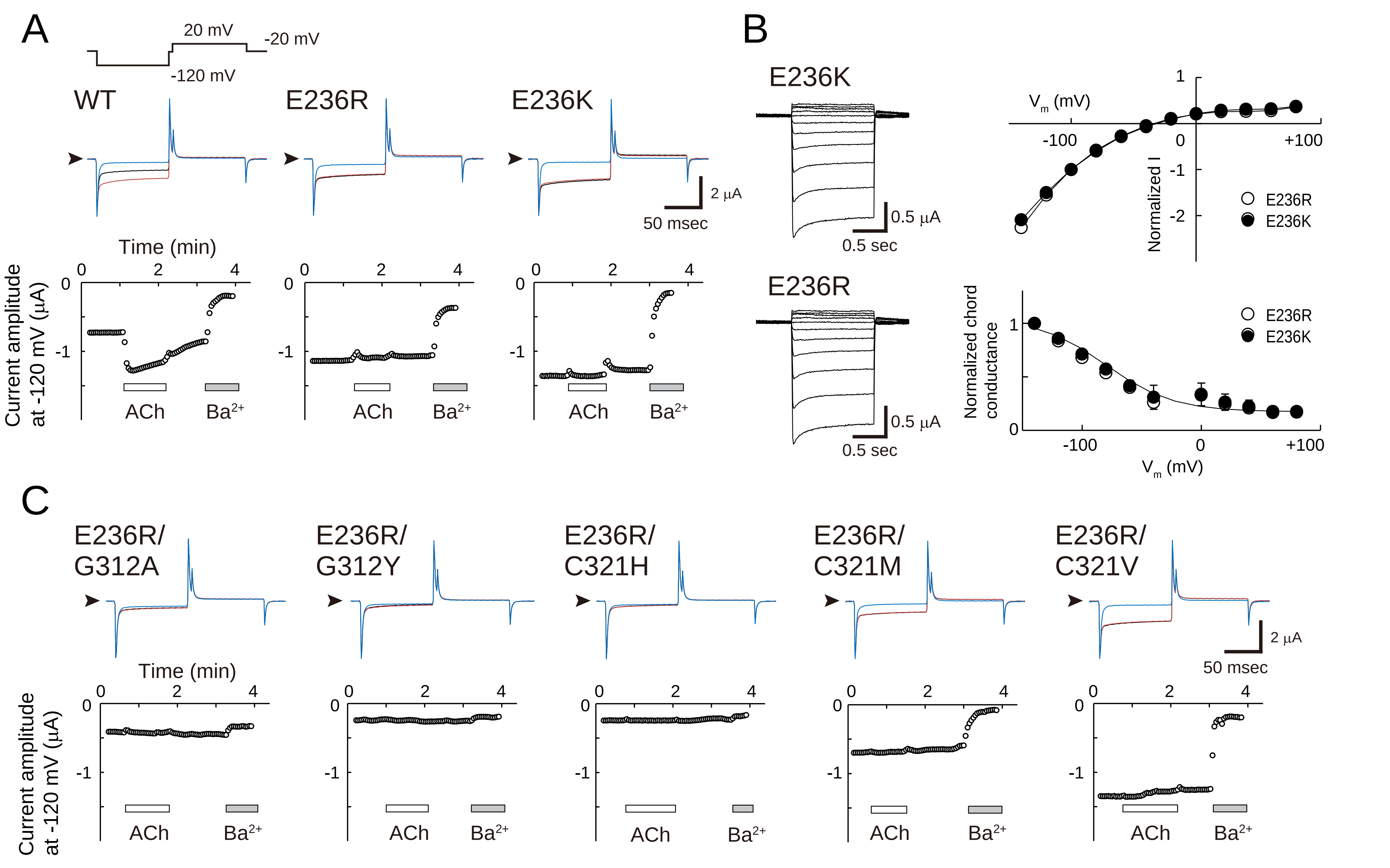

Figure S3. Inanobe et al

IB: aG2A5

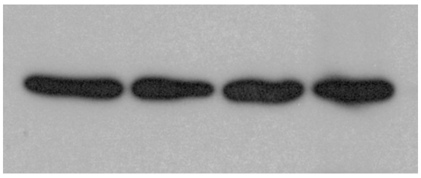

Figure S4. Inanobe et al

Table S1. Measurement of open channel characteristics of Kir3.2 WT and mutants

**Construct**	**Chord conductance**		**Rectification intensity**	
	**$V_{1/2}$ (mV)**	**Slope (mV)**	**(I_{+40}/I_{-80})**	***n***
WT	-34 ± 4	27 ± 3	0.18 ± 0.02	10
E236G	-31 ± 2*	24 ± 5	0.27 ± 0.05	10
E236A	-25 ± 4	23 ± 1	0.16 ± 0.01	8
E236S	-21 ± 3	22 ± 4	0.29 ± 0.03	8
E236D	-58 ± 3*	21 ± 1	0.20 ± 0.03	11
E236N	-69 ± 7*	26 ± 2	0.40 ± 0.01*	5
E236V	N. A.	N. A.	N. A.	
E236Q	-30 ± 2	23 ± 2	0.14 ± 0.02	14
E236R	-78 ± 1*	28 ± 1	0.25 ± 0.03	11
M313G	-38 ± 4	28 ± 1	0.20 ± 0.02	26
M313A	-27 ± 4	30 ± 1	0.16 ± 0.02	15
M313S	-26 ± 2	36 ± 2	0.24 ± 0.01	14
M313V	-73 ± 5*	26 ± 2	0.26 ± 0.02*	10
M313T	-58 ± 5*	29 ± 1	0.32 ± 0.03	13
M313D	-47 ± 2	23 ± 1	0.09 ± 0.01*	12
M313Q	-47 ± 2	21 ± 1	0.09 ± 0.01*	18
M313R	-78 ± 9*	27 ± 2	0.65 ± 0.1*	5

(*n*, number of observation; N.A., not available; *, $p < 0.003$)
